# Integrated Conformational and Lipid-Sensing Regulation of Endosomal ArfGEF BRAG2

**DOI:** 10.1371/journal.pbio.1001652

**Published:** 2013-09-10

**Authors:** Kaheina Aizel, Valérie Biou, Jorge Navaza, Lionel V. Duarte, Valérie Campanacci, Jacqueline Cherfils, Mahel Zeghouf

**Affiliations:** 1 Laboratoire d'Enzymologie et Biochimie Structurales, CNRS, Gif-sur-Yvette, France; 2 Institut de Biologie Structurale, CNRS/CEA/Université Joseph Fourier, Grenoble, France; 3 Unidad de Biofísica CSIC-UPV/EHU, Leioa, Bizkaia, Spain; Princeton University, United States of America

## Abstract

The mechanisms whereby guanine nucleotide exchange factors (GEFs) coordinate their subcellular targeting to their activation of small GTPases remain poorly understood. Here we analyzed how membranes control the efficiency of human BRAG2, an ArfGEF involved in receptor endocytosis, Wnt signaling, and tumor invasion. The crystal structure of an Arf1–BRAG2 complex that mimics a membrane-bound intermediate revealed an atypical PH domain that is constitutively anchored to the catalytic Sec7 domain and interacts with Arf. Combined with the quantitative analysis of BRAG2 exchange activity reconstituted on membranes, we find that this PH domain potentiates nucleotide exchange by about 2,000-fold by cumulative conformational and membrane-targeting contributions. Furthermore, it restricts BRAG2 activity to negatively charged membranes without phosphoinositide specificity, using a positively charged surface peripheral to but excluding the canonical lipid-binding pocket. This suggests a model of BRAG2 regulation along the early endosomal pathway that expands the repertoire of GEF regulatory mechanisms. Notably, it departs from the auto-inhibitory and feedback loop paradigm emerging from studies of SOS and cytohesins. It also uncovers a novel mechanism of unspecific lipid-sensing by PH domains that may allow sustained binding to maturating membranes.

## Abstract 2

The structure of endosomal ArfGEF BRAG2 in complex with Arf1, combined with an analysis of this GEF's efficiency on membranes, reveals a regulatory mechanism that simultaneously optimizes membrane recruitment and nucleotide exchange.

## Author Summary

Understanding the molecular mechanisms that allow guanine exchange factor proteins (GEFs) to coordinate their GDP/GTP exchange activity with their being targeted to specific intracellular membranes is an important issue. In this study, we solved the crystal structure of the ArfGEF BRAG2, an endosomal protein that is involved in invasive phenotypes in various tumors, in a complex with the small GTPase Arf1. We show that the pleckstrin homology (PH) domain of BRAG2 atypically does not auto-inhibit its Sec7 domain (as has been seen in ArfGEFs belonging to the cytohesin family), but instead potentiates nucleotide exchange 10-fold in solution and up to 2,000-fold in the presence of liposomes. This stimulatory effect requires negatively charged membranes, and does not involve a preference of the PH domain for specific phosphoinositides or the use of its canonical lipid-binding pocket. This uncovers a regulatory mechanism in which the PH domain controls GEF efficiency by concurrently optimizing membrane recruitment and nucleotide exchange.

## Introduction

Arf GTPases are pivotal regulators of most aspects of intracellular membrane traffic (reviewed in [1]). They are activated by guanine nucleotide exchange factors (ArfGEFs) that share a conserved Sec7 domain, which stimulates GDP/GTP exchange. Arf GTPases and their GEFs establish intimate interactions with membranes (reviewed in [2]). Arf GTPases feature an allosteric mechanism by which their guanine nucleotide-binding site communicates with their membrane-binding myristoylated N-terminal helix [3], which is harnessed by the Sec7 domain to ensure that their active form is bound to membranes [3],[4]. However, Arf GTPases, notably the most abundant Arf1 isoform, which is found on most membranes of the endocytosis and exocytosis pathways, have little if any membrane specificity on their own. ArfGEFs are therefore predicted to carry elements that restrict their activation of Arf proteins to specific subcellular membranes. Cytohesins are the only ArfGEFs in which such elements have been characterized [5],[6], while the physicochemical and/or curvature properties of membranes that are recognized by other ArfGEF families remain unknown.

BRAG family ArfGEFS (also called IQSec), which are present only in higher organisms, are pivotal regulators of myoblast fusion [7], Wnt signaling [8], and receptor endocytosis [9]–[11] and promote invasive phenotypes in cancer [8],[11]–[13]. Members of this family carry a calmodulin-binding IQ motif in their N-terminus, a Sec7 nucleotide exchange domain followed by a PH domain and a predicted coiled-coil in their C-terminus (reviewed in [14]). BRAG2 (also called GEP100 or IQSec1), the most studied of the three mammalian members, promotes the endocytosis of β1 integrins [9],[10] and of the AMPA receptor in neurons [15], whereas its depletion resulted in increased E-cadherin expression at the cell surface [11],[16]. BRAG2 is responsible for invasive phenotypes in various tumors, notably in breast tumors and lung adenocarcinoma where it binds to tyrosine kinases of the epidermal growth factor receptor (EGFR) family [12] and in melanoma where it is necessary for invasion and metastasis mediated by the Wnt/β-catenin pathway [8]. Current evidence regarding the specificity and the regulation of BRAG2 is fragmentary and somewhat conflicting. BRAG2 has been described as an Arf6-specific GEF in vitro and in transfected cells [9],[10],[12],[17], but also shown to be able to use Arf1 [18] or Arf5 [19] as substrates. It was also proposed to be insensitive to phospholipids [17], or to be specific of phosphatidylinositol 4,5 bisphosphate (PI(4,5)P_2_) [10]. A unique feature that has been put forward is its possible regulation by direct interactions with receptors [12],[13],[15], the mechanism of which is unknown.

Understanding the molecular mechanisms whereby guanine nucleotide exchange factors (GEFs) coordinate their GDP/GTP exchange activities with their targeting to specific intracellular membranes is a major issue in small GTPases biology (reviewed in [2]). Pivotal insight can be gained by reconstituting the activity of GEFs on membranes and capturing them in structures that mimic their soluble and membrane-bound conformations. Such combined studies remain difficult and have been done only for the RasGEF SOS [20],[21]. These pioneering studies and recent investigations of ArfGEFS of the cytohesin [5],[6] and BIG families [22] and of DH-PH containing RhoGEFs of the Lbc family [23],[24] lead to an emerging paradigm in which GEFs are regulated by auto-inhibition combined with a positive feedback loop mediated by freshly produced GTP-bound GTPases. In this schema, the switch from auto-inhibition to full exchange activity is supported by large conformational changes that concurrently optimize nucleotide exchange efficiency and interactions with membranes. Although various other GEFs have been shown to comply with one or another of these mechanisms, notably in the family of DH-PH containing RhoGEFs (reviewed in [2]), the extent to which this scenario can be generalized remains an open issue.

In this study, we investigated the regulatory modalities of BRAG2 on membranes by combined structural and biochemical assays. We find that BRAG2 is regulated by a mechanism that departs considerably from those previously described for other GEFs and involves an atypical PH domain with unprecedented lipid-sensing properties.

## Results

### The Crystal Structure of Arf1–GDP/BRAG2^Sec7-PH^ Reveals an Atypical Membrane-Binding PH Domain

**Figure 1:**
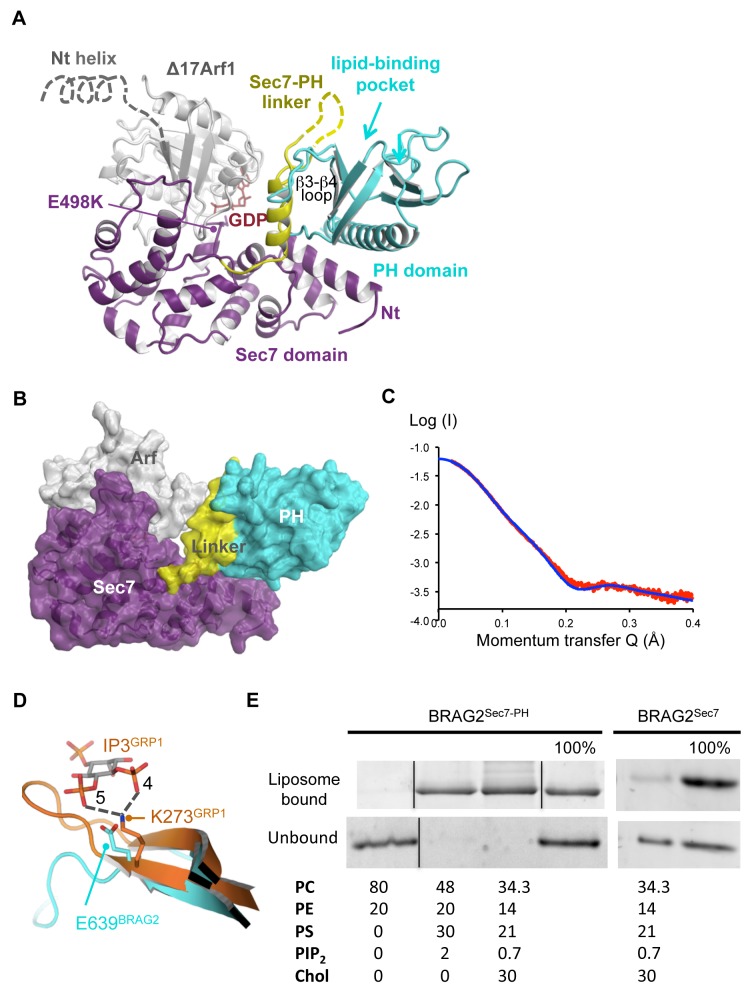
Crystallographic, SAXS, and membrane-binding analysis of BRAG2 reveals an atypical PH domain. (A) Crystal structure of the Δ17Arf1–GDP/BRAG2^Sec7-PH/E498K^ complex (P2 form, crystallographic statistics in Table S1). Arf1 is in grey, and the domains of BRAG2 are color-coded as indicated. Disordered residues in the linker are indicated by a dotted line. The open end of the β-barrel of the PH domain (arrow), which corresponds to the canonical lipid-binding site of PH domains, aligns with the expected position of the membrane-binding myristoylated N-terminal helix of Arf1 (grey dotted line). (B) Surface representation of the Arf1/BRAG2 complex. The linker and the PH domain form a close-packed structure, which establishes a large intramolecular interface with the N-terminus of the Sec7 domain. Arf1 forms edge contacts with the linker. Residues involved in these interfaces are given in Figures S2A and S3. (C) Synchrotron radiation SAXS analysis of unbound BRAG2^Sec7-PH^. Fit of the experimental SAXS data of unbound BRAG2^Sec7-PH^ (red) with the scattering curve calculated from the crystal structure of BRAG2^Sec7-PH^ extracted from the complex (blue) is shown. (D) The PH domain of BRAG2 contains a glutamate that replaces a highly conserved phospholipid-binding lysine. A close-up view of the PH domain of BRAG2 (cyan) superposed on the PH domain of GRP1 bound to IP_3_ (PDB entry code 1U29, orange) is shown. The structure-based sequence alignment of BRAG2 with structures of PH domains with bound phospholipid headgroups is given in Figure S2B. (E) BRAG2^Sec7-PH^ binds to PI(4,5)P_2_-containing liposomes by its PH domain but not to uncharged liposomes. BRAG2^Sec7-PH^ or BRAG2^Sec7^ (1 µM) was submitted to flotation assays using liposomes of the indicated composition (% of 1 mM total lipids). The 100% lane corresponds to the theoretical complete recovery of the protein in the fraction.

BRAG2 proteins carry a Sec7-PH tandem remotely related to that of cytohesins, which are dual Arf1 and Arf6 GEFs [25],[26] and are auto-inhibited by their PH domain in solution [5]. We assessed whether any of these characteristics applies to BRAG2 by measuring its nucleotide exchange activity in solution by tryptophan fluorescence kinetics using recombinant proteins purified to homogeneity (Figure S1A). Arf1 and Arf6 were truncated of their N-terminal helix, which allows them to by-pass the requirement for membranes to be fully activated (reviewed in [27]). BRAG2 constructs encompassing the Sec7 and PH domains and proximal downstream residues (BRAG2^Sec7-PH^, residues 390–763 or 390–811, numbering according to the short isoform BRAG2a [9]) were highly active in solution on both Arf isoforms (k_cat_/K_m_ values in Table 1), suggesting that BRAG2 is not auto-inhibited by its PH domain. We confirmed that BRAG2 has the hallmarks of an Arf1–GEF by showing that a mutant in which the catalytic glutamate was replaced by a lysine (BRAG2^Sec7-PH/E498K^) traps Arf1–GDP in an early intermediate of the exchange reaction (Figure S1B) and that removal of GDP yields the subsequent nucleotide-free Arf/ArfGEF intermediate (Figure S1C). This allowed us to solve the crystal structure of the Arf1–GDP/BRAG2^Sec7-PH/E498K^ complex in two crystal forms (Figure 1A, crystallographic statistics in Table S1). The structure of the complex is similar in the two space groups, but is of better overall quality for the P2 crystal form, which will therefore be used for all subsequent analysis.

**Table 1:** k_cat_/K_m_ of BRAG2 constructs measured in solution using N-terminally truncated Arf proteins and in the presence of liposomes using myristoylated Arf proteins.

k_cat_/K_M_ (10^5^ M^−1^s^−1^)	Δ17Arf1	Δ13Arf6	^myr^Arf1	^myr^Arf6
BRAG2^Sec7^	0.2±0.02	0.1±0.04	0.6±0.04	0.5±0.09
BRAG2^Sec7-PH 390–763^	2.4±0.1	0.5±0.1	390±31	*
BRAG2^Sec7-PH 390–811^	2,5±0.06	0,5±0.1	346±46	*
BRAG2^Sec7-PH/R654E^	2.55±0.01	0.36±0.02	265±20	ND

The two BRAG2^Sec7-PH^ constructs had similar efficiencies, indicating that residues beyond the PH domain are not critical for nucleotide exchange.

* , kinetics for ^myr^Arf6 activation by BRAG2 constructs could not be fitted by a single exponential and were analyzed from initial velocities instead (see text and Figure S4). ND, not done.

The structure reveals that the PH domain of BRAG2 has various unanticipated features, although its fold is similar to those of PH domains of known structures (reviewed in [28]). First, instead of forming an isolated domain, the PH domain is expanded by the linker that bridges the Sec7 and PH domains (residues 592–627), which forms a small subdomain rather than an unstructured tether (Figure 1A and 1B). This subdomain packs against strands β1, β2, and β3 of the PH domain and stabilizes loop β3–β4 away from the pocket that binds phosphoinositides in other PH domains. The interface between the linker and the PH domain (1,200 Å^2^ buried surface area) is largely hydrophobic and contains residues that are highly conserved in the BRAG family (Figures S2A and S3A), indicating that the linker and the PH domain behave as a single domain.

Next, this expanded PH domain establishes a large intramolecular contact with the N-terminus of the Sec7 domain remote from the Arf-binding site (Figures 1B, S2A, and S3B). This contact encompasses the C-terminal helix of the PH domain and proximal downstream residues, which do not form a homodimeric coiled-coil contrary to prediction [9]. Accordingly, BRAG2^Sec7-PH^ behaved as a monomer in solution (Figure S1B and S1C). The interface buries a surface area of 1,800 Å^2^, suggesting that it is a constitutive rather than a regulatory intramolecular interaction. To assess whether this interaction exists in unbound BRAG2, we analyzed the conformation of BRAG2 in solution by synchrotron small-angle X-ray scattering (SAXS). The SAXS curve calculated from the structure of BRAG2^Sec7-PH^ extracted from the crystalline complex agreed well with the experimental SAXS curve of unbound BRAG2 in solution (Figure 1C). These observations, together with the fact that BRAG2 is not auto-inhibited, suggest that the predominant conformation of unbound BRAG2^Sec7-PH^ is similar to that seen in the crystalline Arf1–BRAG2 complex. Accordingly, the expanded PH domain is not auto-inhibitory and does not move away to activate Arf proteins. Given the structural conservation of the Sec7 domain, we surmise that its N-terminus may serve an as yet underestimated purpose in scaffolding intramolecular interactions in other ArfGEF families, which may explain why mutations in this region impaired plant Golgi ArfGEFs functions [29].

Finally, the PH domain of BRAG2 displays a striking sequence difference with phosphoinositide-specific PH domains: Glu639 in strand β1 replaces a highly conserved lysine in the canonical lipid-binding pocket (as reviewed in [28]) (Figures 1D and S2B). This lysine is critical for PI(4,5)P_2_ recognition, as exemplified in cytohesins where its mutation to an alanine abolished the GEF activity on membranes [6]. The glutamate in BRAG2 would thus be predicted to generate repulsive interactions that impair PI(4,5)P_2_ binding. We analyzed the binding of BRAG2 to PI(4,5)P_2_-containing liposomes by a flotation assay, which was preferred over a co-sedimentation assay for its ability to accurately separate liposome-bound proteins from insoluble misfolded proteins. We observed significant binding to liposomes containing PS as the sole negatively charged lipid (Figure S1D) and near complete binding with liposomes containing PS and PI(4,5)P_2_ whether or not complemented with cholesterol, a major component that distinguishes the plasma membrane from other cellular membranes (Figures S1D and 1E). Binding was dependent on both the expanded PH domain and on negatively charged lipids, as no binding was detected with the Sec7 domain alone (residues 390–594) or with uncharged lipids (Figure 1E). Thus, the atypical glutamate does not prevent the PH domain of BRAG2 from binding to membranes.

### BRAG2 Is Regulated by Combined Conformational and Membrane-Controlled Contributions

**Figure 2:**
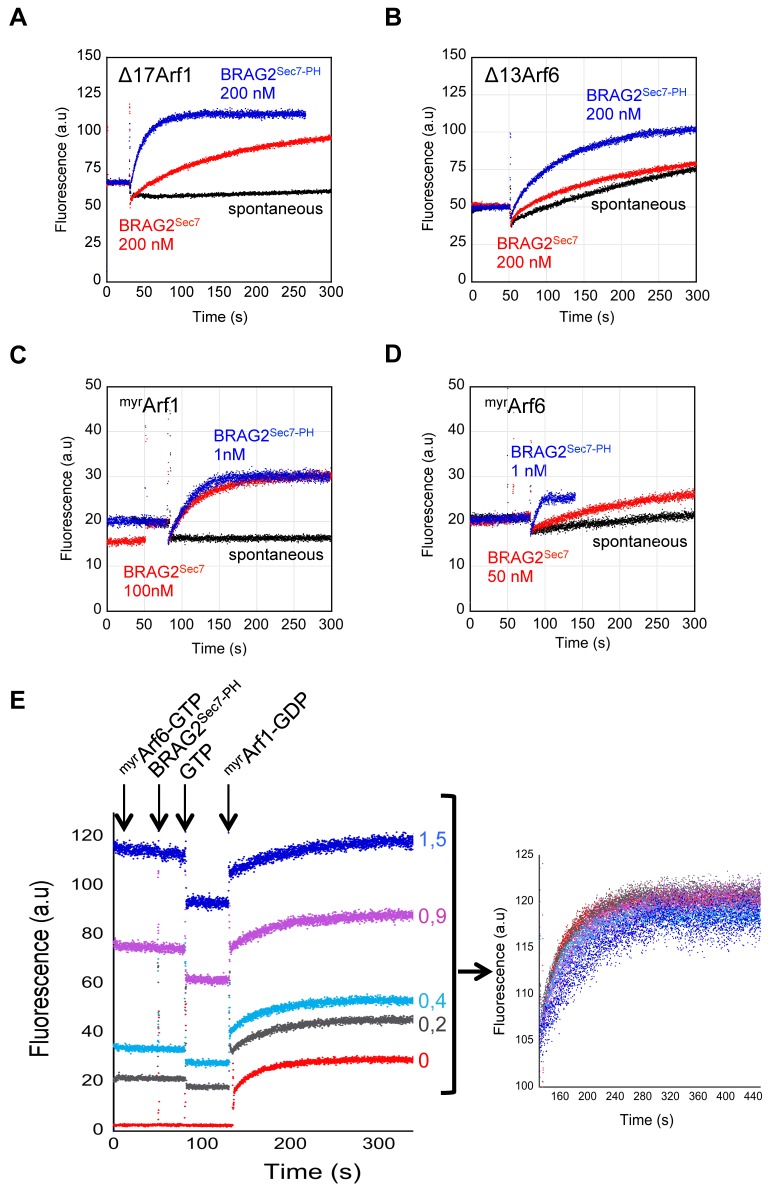
Quantitative analysis of BRAG2 nucleotide exchange efficiency reveals a dual Arf1/Arf6 specificity and the potentiating role of the PH domain. (A and B) BRAG2 activates Arf1 and Arf6 and is potentiated by its PH domain in solution. Representative tryptophan fluorescence kinetics curves used to determine k_cat_/K_m_ given in Table 1 are shown. Exchange reactions were done with 1 µM truncated Arf proteins. SDS-PAGE gels of the proteins are shown in Figure S1A. (C and D) BRAG2 exchange activity towards Arf1 and Arf6 is strongly potentiated by membranes. Representative tryptophan fluorescence kinetics used to determine k_cat_/K_m_ values given in Table 1 are shown. Exchange reactions were done with 100 µM liposomes (34.3% PC, 14% PE, 21% PS, 0,7%PI(4,5)P_2_, 30% cholesterol) and with 0.4 µM ^myr^Arf proteins. The detailed analysis of Arf6 activation using experimental initial velocities is given in Figure S4. (E) BRAG2 is not regulated by a feedback loop. Activation of ^myr^Arf1 by BRAG2^Sec7-PH^ was analyzed by tryptophan fluorescence kinetics using the same liposomes as in Figure 2C. Liposomes were pre-incubated with increasing amounts of ^myr^Arf6–GTP as indicated. The right panel shows the kinetics associated with the formation of ^myr^Arf1–GTP corrected for the intrinsic fluorescence of ^myr^Arf6–GTP.

The crystal structure of the Arf1–GDP/BRAG2^Sec7-PH^ complex captured the relative arrangement between Arf1, the catalytic Sec7 domain, and the PH domain in the course of the exchange reaction. First, it shows that Arf1 forms edge contacts with the PH domain. The interface involves the switch 1 of Arf1 and the Sec7-PH linker subdomain and is loosely packed (250–450 Å^2^ buried surface area, Figures 1A, 1B, S1E, S2A, and S3C). To analyze whether this contact contributes to the efficiency of the exchange reaction, we compared the exchange rates of BRAG2^Sec7^ and BRAG2^Sec7-PH^ in solution. BRAG2^Sec7-PH^ was 10 times more active than BRAG2^Sec7^ towards Arf1, and 4 times more active towards Arf6 (k_cat_/K_m_ values in Table 1, Figure 2A and 2B). Thus, the conformation of the Sec7-PH linker as a small domain rather than as an extended tether allows the enlarged PH domain to potentiate the exchange reaction, a contribution that we therefore call “conformational.” The loose packing of the Arf/PH domain contact probably allows for the rotation of Arf towards the catalytic site that occurs as the exchange reaction proceeds [4],[30] and for the subsequent release of Arf–GTP.

Next, the structure shows that BRAG2-bound Arf1–GDP has undergone a two-residue shift of the interswitch, a conformational change that has been shown to occur prior to GDP dissociation [4] and to secure active Arf proteins to membranes ([30], reviewed in [3]), suggesting that the complex mimics a membrane-bound intermediate of the exchange reaction. The intramolecular interaction between the Sec7 domain and the enlarged PH domain constrains the relative orientations of Arf and the PH domain, thereby aligning the membrane-binding N-terminus of Arf1 and the PH domain on the same side of the complex (Figure 1A). They could thus bind to membranes simultaneously, potentially contributing to BRAG2 efficiency. This was analyzed by reconstituting the exchange reaction on liposomes (Figure 2C and 2D) using highly pure myristoylated Arf1 and Arf6 (Figure S1A). The efficiency of BRAG2^Sec7-PH^ towards Arf1 on liposomes was increased by 160-fold compared to its efficiency in solution (k_cat_/K_m_ values in Table 1). Liposomes did not increase the exchange efficiency of BRAG2^Sec7^ (Table 1), indicating that the effect requires the PH domain. BRAG2^Sec7-PH^ also strongly activated ^myr^Arf6 in the presence of liposomes, although with unusual kinetics that could not be analyzed by a single exponential fit and were analyzed using initial velocities (Vi) (Figures 2D, S4A and S4B). Vi values were linear as a function of BRAG2 concentration and were in the same range as those found for Arf1 (Figure S4C), indicating that membranes potentiate the efficiency of BRAG2^Sec7-PH^ towards Arf1 and Arf6 to the same extent. Altogether, these observations reveal that membranes strongly potentiate the efficiency of BRAG2, and that this effect depends on the unconventional PH domain.

Regulation of ArfGEFs on membranes by a positive feedback loop mediated by freshly produced Arf–GTP has been put forward for plasma membrane cytohesins [6] and Golgi BIG [22]. Feedback loops can be highlighted *in vitro* by preloading liposomes with increasing amounts of Arf–GTP prior to measuring nucleotide exchange rates. A positive feedback loop would then be detected by an increase of the exchange rates, while a decrease would indicate a negative feedback loop. The exchange rates of BRAG2^Sec7-PH^ towards ^myr^Arf1 were unaffected when increasing amounts of ^myr^Arf6–GTP were pre-loaded on liposomes (Figure 2E). Thus, BRAG2 is not regulated by a feedback loop, unlike cytohesins and BIG.

### Unspecific Recognition Of Negatively Charged Membranes by the PH Domain Outside Its Canonical Lipid-Binding Pocket

**Figure 3:**
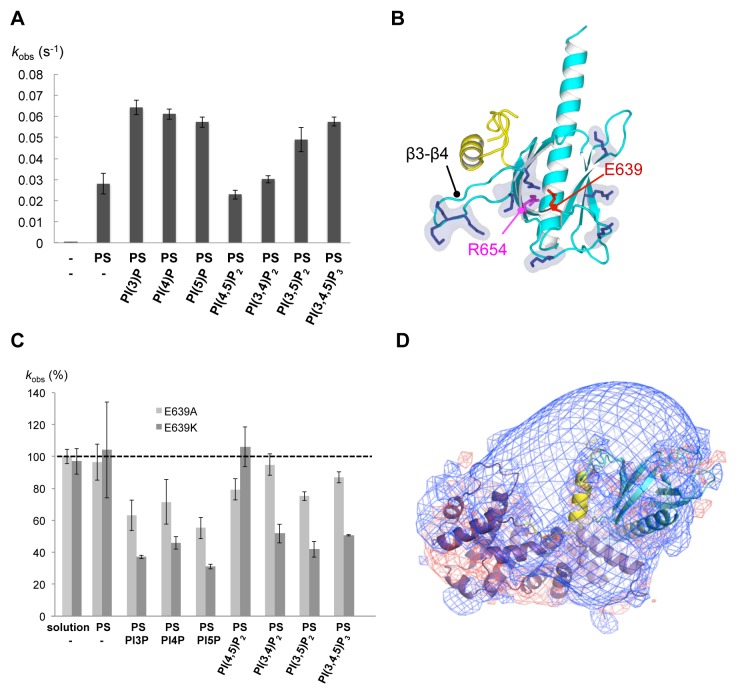
Unspecific sensitivity of the atypical PH domain of BRAG2 to negatively charged membranes. (A) BRAG2 is activated by negatively charged membranes but does not discriminate between phosphoinositides. The histogram shows nucleotide exchange rates of BRAG2^Sec7-PH^ (1 nM) towards ^myr^Arf1–GDP (0.4 µM) using 100 µM of liposomes containing 2% PI and 30% PS complemented with 48% PC and 20% PE, except for uncharged liposomes containing 80% PC and 20% PE. Reactions were initiated by addition of 100 µM GTP. *k*_obs_ values are means of at least three experiments and are given ±S.D. (B) The proposed membrane-binding surface of the PH domain of BRAG2. Positively charged residues are shown in dark blue. Residues mutated in the canonical lipid-binding pocket are shown. (C) BRAG2 does not use the lipid-binding pocket of its PH domain to recognize negatively charged membranes. Nucleotide exchange activity of BRAG2^Sec7-PH^ mutants carrying the E639A and E639K mutation in the PH pocket, using ^myr^Arf1 and liposomes of the indicated compositions. *k*_obs_ are expressed as a percentage of the exchange rate of wild-type BRAG2^Sec7-PH^. Nucleotide exchange in solution using Δ17Arf1–GDP is shown on the left. (D) The proposed membrane-facing surface of the PH domain has a strong positive electrostatic potential. The electrostatic potential map is contoured at –5 kT/e (in red) and 5 kT/e (blue). The view as in Figure 1A.

Most phosphatidylinositides (PIs) (reviewed in [28]) as well as phosphatidylserine (PS) [31] can be recognized by specific PH domains. Since the unusual glutamate located in the lipid pocket of the PH domain did not preclude BRAG2 from binding to PI(4,5)P_2_-containing liposomes or from activating Arf proteins on these liposomes, we investigated whether it could serve as a sentry to exclude other PIs. We took advantage of the sensitivity of the nucleotide exchange kinetics assay to compare the seven major PIs (PI(3)P, PI(4)P, PI(5)P, PI(3,4)P_2_, PI(3,5)P_2_, PI(4,5)P_2_, and PI(3,4,5)P_3_). Surprisingly, none of these phosphoinositides significantly increased or decreased the nucleotide exchange rate of BRAG2^Sec7-PH^ towards ^myr^Arf1 taking PI(4,5)P_2_-containing liposomes as a reference (maximum 2-fold) (Figure 3A). A nucleotide exchange rate in the same range was achieved when PS (10–30%) was the sole negatively charged lipid added to liposomes. In contrast, the activity of BRAG2^Sec7-PH^ was weak and remained in the same range as that of BRAG2^Sec7^ with liposomes devoid of negatively charged lipids. These data indicate that the PH domain of BRAG2 is sensitive to negatively charged membranes but does not discriminate between the different PIs. Notably, it is not specific for PI(4,5)P_2_ unlike previously suggested [10]. Consistently, we did not detect binding of IP_3_, the soluble headgroup of PI(4,5)P_2_, to BRAG2 ^Sec7-PH^ as measured by isothermal calorimetry, unlike what would have been expected for a tight specific interaction.

These observations suggest that the PH domain of BRAG2 may not use its canonical lipid-binding pocket to recognize negatively charged lipids. We analyzed the contribution of this pocket by mutating Arg654, a highly conserved residue located at the bottom of this pocket where it binds PI phosphates in PI-specific PH domains (Figures 3B and S2B). The R654E charge reversal mutation had no effect of nucleotide exchange efficiency on membranes containing PS and PI(4,5)P_2_ (k_cat_/K_m_ values in Table 1), supporting the hypothesis that the pocket is not involved in membrane recognition. To analyze whether Glu639 is the sole residue responsible for the lack of phosphoinositide specificity and/or recognition, we analyzed the exchange rates of BRAG2^Sec7-PH^ constructs carrying the E639A or E639K mutations in the presence of liposomes containing each of the different PIs (Figure 3C). Neither of the mutations had a marked effect on nucleotide exchange (maximum 2-fold decrease) and they had no effect when assayed in the presence of liposomes containing PS as the sole negatively charged lipid or containing PI(4,5)P_2_. Notably, the E639K mutation did not restore phosphatidylinositide specificity but slightly inhibited nucleotide exchange. These data indicate that the atypical glutamate is not the only feature responsible for the lack of specificity of BRAG2 for PIs. The periphery of the canonical lipid-binding pocket in BRAG2 is enriched in positively charged residues (Figure 3B), resulting in a highly positive electrostatic potential (Figure 3D). The linker subdomain contributes to organizing this positively charged patch by stabilizing the loop β3–β4, which contains several conserved lysines, away from the pocket (Figures 1A and S2B). We propose that BRAG2 uses this positively charged surface to establish nonspecific electrostatic interactions with the phosphates of PS and PIs, rather than recognizes specifically any of them by the canonical pocket.

### Structural Basis for Divergent Regulation of BRAG and Cytohesin ArfGEFs

**Figure 4:**
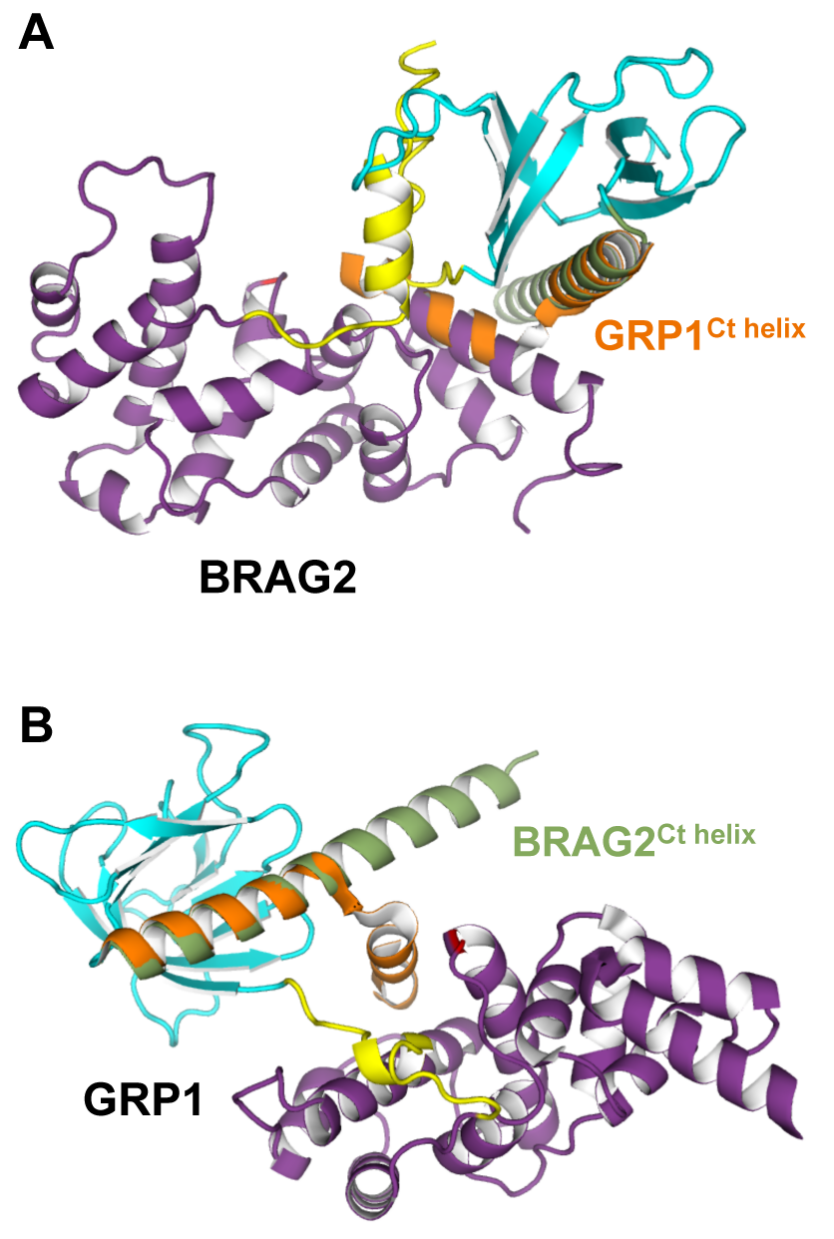
Structural basis for the diverging regulatory mechanisms of BRAG2 and cytohesins. (A) Superposition of the PH domain of GRP1 to that of BRAG2 shows that the kinked auto-inhibitory C-terminal helix of GRP1 (in orange) would conflict with the Sec7 domain of BRAG2. (B) Superposition of the PH domain of BRAG2 to the auto-inhibited structure of GRP1 shows that the straight C-terminal helix of BRAG2 (in green) would not be auto-inhibitory in GRP1.

ArfGEFs of the cytohesin family are regulated by a positive feedback loop mediated by their PH domain, which switches from auto-inhibition of the Sec7 domain in solution [5] to an activating role on membranes by coincident binding to PI(4,5)P_2_ or PI(3,4,5)P_3_ phosphoinositides [32] and to GTP-bound Arf proteins [6]. Cytohesins and BRAG ArfGEFs have a closely related organization encompassing a Sec7 and PH domain in tandem, which would predict that they have similar regulatory modes. At odds with this prediction, our study reveals that BRAG2 is not auto-inhibited by its PH domain, is not regulated by a feedback loop, and does not respond to specific phosphoinositides. We find that unanticipated differences between the structures of cytohesins and BRAG explain their diverging mechanisms. First, elements proximal to the PH domain that insert into the Sec7 active site to mediate auto-inhibition in cytohesins [5] have a different structure in BRAG2, where they support a constitutively active conformation instead. Notably the unusually long C-terminal helix of the PH domain is kinked in cytohesins, and hence would conflict with the N-terminus of the Sec7 domain in BRAG2 (Figure 4A), whereas it is straight in BRAG2 and would not be autoinhibitory in cytohesins (Figure 4B). Next, the Sec7-PH linker in BRAG2, by behaving as a subdomain that enlarges the PH domain (Figure 1A and 1B), shields the surface of the PH domain predicted to bind Arf–GTP in cytohesins (Y290 and I303 corresponding to V664 and S683 in BRAG2) [6],[26] and hence makes it unavailable for feedback regulation. This also implies that cytohesins cannot adopt the same active conformation as BRAG2, which would not be compatible with their binding of Arf–GTP. Finally, differences in sequence and conformation in and near the canonical lipid-binding pocket of the PH domain explain why cytohesins recognize PI(4,5)P_2_ or PI(3,4,5)P_3_ phosphoinositides specifically, while BRAG2 recognizes negatively charged membranes nonspecifically without using its pocket (Figure 1D and S2B). Notably, stabilization of the long β3–β4 loop of the PH domain by the linker in BRAG2 organizes a positively charged surface that accounts well for its unspecific avidity for negatively charged lipids (Figure 3B). Thus, localized differences between these related ArfGEFs add up to yield considerably different regulatory regimes, which could not be predicted from their overall domain homologies alone.

## Discussion

### The Integrated Conformational and Lipid-Sensing Regulation of BRAG2 Expands the Repertoire of GEF Regulatory Mechanisms

Understanding how small GTPases and their regulators depend on their lipid environment for their activity and specificity is a major issue in small GTPases biology that remains poorly understood. In this study, we combined structural analysis and nucleotide exchange reconstituted on liposomes to analyze how the ArfGEF activity of endosomal and cancer-involved BRAG2 is regulated on membranes. Our data reveal that the structure of BRAG2 constrains the relative orientations of its catalytic Sec7 domain, of its atypical membrane-binding PH domain, and of Arf such as to optimize them concurrently for membrane recruitment and for nucleotide exchange. The PH domain plays a pivotal role in modulating BRAG2 nucleotide exchange efficiency by integrating two separable components. On the one hand, its extension by the Sec7-PH linker allows it to form a loose interaction with Arf GTPases, thus providing a conformational contribution to the exchange efficiency of BRAG2^Sec7-PH^ by about one order of magnitude compared to the Sec7 domain alone in the absence of membranes. On the other hand, it increases the exchange efficiency of BRAG2^Sec7-PH^ by about two orders of magnitude by a dual membrane-controlled spatial contribution comprised of (1) an atypical interaction with negatively charged membranes outside the canonical lipid-binding pocket (Figure 3B) and (2) an intramolecular interaction with the Sec7 domain that increases the probability of a catalytically productive encounter between Arf and BRAG by aligning their lipid-binding regions (Figure 1A). Remarkably, the conformational and spatial contributions are cumulative, resulting in a 2,000-fold increase of nucleotide exchange efficiency between BRAG2^Sec7^ in solution and BRAG2^Sec7-PH^ on membranes (Table 1). Other members of the BRAG ArfGEF subfamily are highly homologous to members of the BRAG2 subgroup in the regions involved in lipid binding and nucleotide exchange. Notably, residues involved in intramolecular linker/PH and PH/Sec7 interactions (Figure S2A) and positively charged residues at the periphery of the canonical lipid-binding pocket () are highly conserved in the entire subfamily. The only significant difference is a 11-residue insert in the BRAG2 linker, which is highly flexible in our structures and does not carry positively charged residues, making it unlikely that is has a major conformational or lipid-binding contributions. We therefore propose that the regulatory modalities of other BRAG members are similar to those of BRAG2.

The modalities of this large potentiation of the intrinsic activity of a GEF domain by a noncatalytic domain depart from the emerging paradigm of up-regulation of Ras, Arf, and Rho GEFs by auto-inhibition release via positive feedback loops [5],[6],[20]–[24]. The mechanism of BRAG2 thus reveals that not all GEFs comply to the feedback regulatory paradigm and expands the repertoire of mechanisms that should be considered in future studies of GEFs.

### Fine-Tuning of the Production of Activated Arf Proteins in Time and Space by ArfGEFs

**Figure 5:**
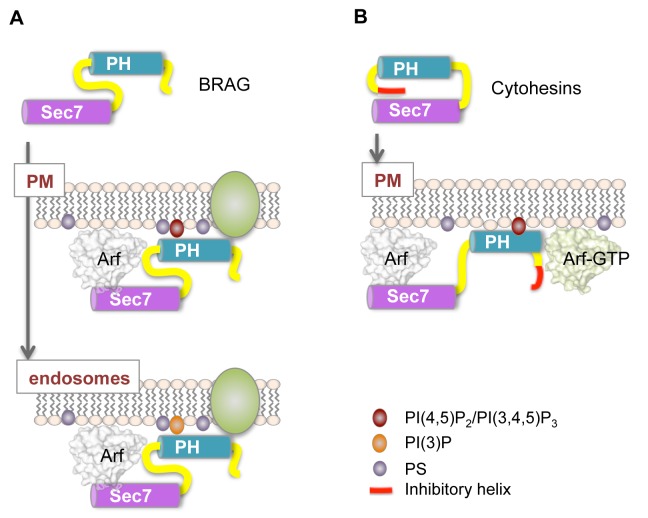
Diverging regulatory models of cytohesin and BRAG ArfGEFs on cellular membranes. (A) BRAG2 is constitutively active in solution (top panel), but strongly potentiated by negatively charged membranes such as those found at the plasma membrane (middle panel) and early endosomes (bottom panel). The PH domain interacts nonspecifically with PS- and PI-containing membranes outside the canonical lipid-binding pocket. Additional specificity may be achieved by interaction with receptors (shown in green). (B) Cytohesins are autoinhibited in solution (top panel) and activated by specific binding of their PH domain to PI(4,5)P_2_ or PI(3,4,5)P_3_ and to Arf–GTP at the plasma membrane (bottom panel).

Although it is known that many PH domains do not bind PIs with high specificity (reviewed in [28]), the PH domain of BRAG2 is, to the best of our knowledge, the first PH domain shown to use nonspecific recognition of negatively charged membranes to quantitatively control a biochemical activity. An important issue arising is thus why BRAG2 activity would depend on the unspecific recognition of PS and PI-containing membranes. Different PIs in combination with PS constitute major signposts of plasma and endocytic membranes (reviewed in [33],[34]). PI(4,5)P_2_, PI(3,4,5)P_3_, as well as PI(4)P to some extent [35] contribute to define plasma membrane identity, while PI(3)P [36] and PI(5)P [37] are preferentially found on early endosomes. On the other hand, PS is the predominant anionic lipid at the plasma membrane and a major lipid in early endosomal membranes where it contributes to target or maintain proteins, but it is poorly abundant on late endosomes and on Golgi membranes [38]. This suggests an appealing model in which the PH domain of BRAG2 would be tailored for dual and/or sustained interaction with both plasma and early endosomal membranes. This could allow BRAG2 to activate Arf proteins at the plasma membrane where receptors nearing endocytosis are located, and to remain active on maturating membranes entering the receptor endocytic pathway (Figure 5A). Divergences in regulation between cytohesin and BRAG ArfGEFs highlighted in this study may thus reflect their adaptation to distinct functional needs. Autoinhibition and PI specificity of cytohesins would allow them to be temporally and spatially restricted by phosphoinositide signals at the plasma membrane (Figure 5B). BRAG, in contrast, would be suited for sustained activity on membranes undergoing phospholipid maturation along the receptor endocytosis pathway (Figure 5A). Future work will be needed to analyze whether the efficient regulatory mechanism of BRAG2 relies either on autoregulatory features mediated by N-terminal elements of BRAG2 and/or on direct interaction with receptors. The dual specificity of cytohesins and BRAG2 for Arf1 and Arf6 could also fulfill different functional needs. While in cytohesins it may amplify an initial Arf signal, in BRAG2 it could reflect the sequential and/or simultaneous activation of different Arf isoforms. This could explain why, while BRAG2 has been consistently shown to activate Arf6, its depletion and that of Arf6 have opposite effects on endocytosis of β1 integrins [9], or that both Arf1 and Arf6 regulate the Wnt/β-catenin pathway [39], a pathway that was recently demonstrated to require BRAG2 [8]. The robust structural and biochemical characterization of BRAG2 regulation reported in our study should now be valuable for future investigations of the coordination between trafficking pathways and receptor endocytosis and signaling in normal and cancer cells.

## Materials and Methods

### Protein Expression and Purification

PCR products encoding human BRAG2^Sec7^ (residues 390–594) or BRAG2^Sec7-PH^ (residues 390–763 or 390–811) were cloned into the pProEX-HTb vector (Invitrogen) as a fusion with a N-terminal 6-His tag followed by a tobacco etch virus (TEV) protease cleavage site. BRAG2 mutants were generated with the QuikChange II XL kit (Stratagene). All constructs were confirmed by sequencing. All BRAG2 constructs were expressed in *E. coli* BL21 Gold strain at 37°C with 3 h of induction with IPTG (0.5 mM). Seleno-methionine (SeMet) BRAG2^Sec7-PH/E498K^ was incorporated as described in [40]. Cells were disrupted by sonication in buffer A (20 mM phosphate buffer pH 7.4, 10 mM imidazole, 500 mM NaCl, and 5 mM β-mercaptoethanol) completed with 0.5 mg/ml of lysozyme and a protease inhibitor cocktail. Cleared lysates were loaded on nickel-nitrilotriacetic acid (Ni-NTA) affinity chromatography (HisTrap FF, GE Healthcare) equilibrated with buffer A, eluted with a 10–500 mM linear imidazole gradient, and when indicated, cleaved with the TEV protease (1∶10 w/w) overnight at 4°C and reloaded on a HisTrap column. For BRAG2^Sec7-PH/E498K^, an additional step of ion exchange chromatography was performed on a MonoS column (GE Healthcare). Purification of all BRAG2 constructs was polished by gel filtration on a Superdex 75 XK 16/90 (GE Healthcare) equilibrated with 20 mM HEPES pH 7.4, 5 mM β-mercaptoethanol, and 100–500 mM NaCl. Human Δ17Arf1 and Δ13Arf6 were expressed and purified as described in [41] and [42] and loaded with GDP prior to kinetics experiments. Nucleotide content was assessed by thermal denaturation followed by ion exchange chromatography. Myristoylation of full-length Arf1 was done by co-expression with yeast N-myristoyl transferase and purified as described in [43]. Myristoylation of full-length Arf6 carrying a C-terminal 6-His tag was done *in vitro* with recombinant human N-myristoyltransferase [44]. SDS-PAGE gels of proteins used in this study are shown in Figure S1A.

### Preparation of the Arf1/BRAG2 Complexes

The Δ17Arf1–GDP/BRAG2^Sec7-PH/E498K^ complex was obtained by incubation in 20 mM HEPES pH 7.4, 100 mM NaCl, 1 mM MgCl_2_, 5 mM β-mercaptoethanol, and 2 mM EDTA. The nucleotide-free complex was obtained by incubating Δ17Arf1–GDP and BRAG2^Sec7-PH^ (2∶1 ratio) with 1 U/mg of alkaline phosphatase (Sigma) in 20 mM HEPES pH 7.4, 150 mM NaCl, 4 mM β-mercaptoethanol overnight at 4°C. Both complexes were purified by size exclusion chromatography on a Superdex75 10/300 column (GE Healthcare) equilibrated with their incubation buffer, supplemented with 5 mM EDTA for the nucleotide-free complex.

### Liposome Preparation and Flotation Assay

All lipids were from Avanti Polar Lipids, and NBD-PE was from Invitrogen. Liposomes were prepared as described [6] in 50 mM HEPES pH 7.4, 120 mM potassium acetate buffer, and freshly extruded through a 200 nm filter (Whatman). Liposome flotation assays were performed as described in [45]. Briefly, 1 µM of protein was incubated with liposomes (1 mM total lipids) for 5 min at room temperature in 50 mM HEPES pH 7.4 buffer containing 120 mM potassium acetate, 1 mM MgCl_2_, and 1 mM DTT (HKM buffer). The solution was brought to 30% sucrose, overlaid with two layers of HKM containing 25%, and no sucrose then submitted to centrifugation at 240,000 *g* in a TLS55 swing rotor (Beckman) for 1 h at 20°C. Liposome-bound proteins (top fraction) and unbound proteins (bottom fraction) were collected manually and analyzed by SDS-PAGE after SYPRO Orange (Invitrogen) staining using a Fuji LAS-3000 fluorescence imaging system. All experiments were done in triplicate.

### Nucleotide Exchange Assays

Nucleotide exchange kinetics were monitored by tryptophan fluorescence with excitation and emission wavelengths of 292 nm and 340 nm on a Cary Eclipse fluorimeter (Varian) under stirring. All experiments were carried out at 37°C by the successive addition of Arf, BRAG2, and finally 100 µM GTP to initiate nucleotide exchange. Exchange assays without liposomes were performed in 50 mM HEPES pH 7.4, 50 mM NaCl, 2 mM MgCl_2_, 2 mM β-mercaptoethanol, using 1 µM Arf and BRAG2 constructs (0–0.4 µM range) for catalytic efficiency (k_cat_/K_m_) determinations. Exchange assays with liposomes were done with 100 µM pre-warmed liposomes in 50 mM HEPES pH 7.4, 120 mM potassium acetate, 1 mM MgCl_2_, 1 mM DTT with 0.4 µM ^myr^Arf and BRAG2 constructs (0–1 nM range) for k_cat_/K_m_ determinations, or a fixed concentration of 1 nM for single exchange rates (k_obs_) determination. Except for ^myr^Arf6 activation, k_obs_ were determined from a monoexponential fit taking into account the linear drift of fluorescence due to photobleaching. k_cat_/K_m_ were obtained following a Michaelis-Menten formalism as described in [41] from:
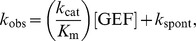
where *k*_spont_ is the spontaneous nucleotide exchange rate constant. All experiments were done at least in triplicate. ^myr^Arf6 activation kinetics could not be analyzed by a single exponential fit. This unusual behavior was observed whether the exchange reaction was monitored by tryptophan fluorescence, which measures Arf conformational change upon nucleotide exchange (Figure S4A), or by mantGTP fluorescence, which measures nucleotide exchange directly (unpublished data). This behavior was seen with other Arf6–GEFs ([46], our unpublished results), but was not observed with BRAG2^Sec7^ in the presence of liposomes or with BRAG2^Sec7–PH^ in solution, and was not due to undesirable liposome aggregation due to Arf6 or to BRAG2 (Figure S4B). This behavior was also independent of the concentration of ^myr^Arf6 used in the assay, thus ruling out a saturation effect (unpublished data). We surmise that it is due to the fact that Arf6 releases GDP spontaneously much faster than Arf1 ([47], compare also Figure 2A to 2B and 2C to 2D), resulting in a fraction of membrane-bound nucleotide-free ^myr^Arf6–GDP that undergoes fast activation. To circumvent this feature, Arf6 exchange kinetics were analyzed using initial velocities (Vi), which were plotted as a function of BRAG2 concentration.

### Feedback Loop Experiment

Liposomes (150 µM) were loaded with increasing amounts of ^myr^Arf6–GTP before 1 nM BRAG2^Sec7-PH^, 100 µM GTP, and 0.4 µM ^myr^Arf1–GDP were added in sequence. The exchange rate of ^myr^Arf1 was determined by fitting the fluorescence change of the second part of the reaction to a single exponential.

### Crystallization and Structure Determination

The BRAG2^Sec7-PH/E498K^/Δ17Arf1–GDP complex was concentrated to about 1.5 mg/ml for crystallization. Crystals were obtained either with Se-Met BRAG2 with the 6-His tag cleaved, and with native BRAG2 carrying the tag (native crystals). Se-Met crystals grew in 0.15 M ammonium sulfate, 0.1 M MES pH 6, and 16% PEG 4000, and native crystals in 0.15 M ammonium sulfate, 0.1 M NaH_2_PO_4_/Na_2_HPO_4_ pH 6, and 13% PEG 4000. Crystals were transferred to the reservoir solution adjusted at 17% PEG 4000 and supplemented with 20% PEG 400 and flash frozen in liquid nitrogen. Diffraction data were collected at beamline PROXIMA1 (SOLEIL Synchrotron, Gif-sur-Yvette, France) at 0.98 Å wavelength for the native crystals, and at the f′ maximum of the selenium edge (0.979 Å) for the Se-Met crystals. Intensities were integrated and scaled with XDS [48] for the Se-Met crystals and integrated with imosflm [49] and scaled with scala for the native crystal. The native crystals belong to space group C2 and contain two complexes related by translational non-crystallographic symmetry (TNCS) in the asymmetric unit, and the Se-Met crystals belong to space group P2 and contain four complexes related by TNCS in the asymmetric unit.

The selenium anomalous signal from the Se-Met crystals did not allow for phasing. Alternatively, the structure of the C2 native crystal was solved by molecular replacement with the program AMORE [50], using Δ17Arf1–GDP from the Δ17Arf1–GDP/ARNO complex (PDB entry 1R8S, [4]), the Sec7 domain from the Δ17Arf1–GDP/ARNO (PDB entry 1R8S) from which sequence differences were modeled as alanines, and the PH domain of BRAG2 (unpublished PDB entry 3QWM) as search models. The solution was found using TNCS with data between 15 and 4.5 Å. A similar strategy using TNCS and data between 45 and 3.5 Å was used to solve the P2 crystal form. Rigid body refinement was done with Phaser [51]. Refinement was carried out with Phenix [52] and autoBUSTER [53], in alternation with graphical building using Coot [54]. The bound nucleotide is GDP-3′P, a GDP derivative produced by *E. coli* under stress conditions that commonly substitutes for GDP in other small GTPases structures without impairing their structures (PDB entries 2HXS, 2ZJ6, 1R8Q, 1MR3). The conformation of Arf1 and its position relative to the Sec7 domain of BRAG2 are also similar to those previously observed for Arf1–GDP in complex with the Sec7 domain of ARNO carrying the E/K mutation [4], indicating that it is not due to GDP-3′-P. Crystallographic statistics and details of the refinement procedure are given in Table S1. Coordinates have been deposited with the Protein Data Bank with accession code 4C0A. The electrostatic potential was calculated from the crystallographic coordinates of BRAG2 with PDB2PQR [55]. Contour levels were expressed as multiples of dimentionless unit kT/e, where k is the Boltzmann's constant, T is the temperature, and e is the charge of an electron, and were displayed with PYMOL.

### Small Angle X-Ray Scattering

SAXS experiments were conducted on beamline SWING (SOLEIL Synchrotron, Gif-sur-Yvette, France) essentially as described in [56]. The histidine tag of BRAG2^Sec7-PH^ was cleaved for SAXS data collection, as unstructured tags add noise to SAXS experiments. The protein sample was injected into a size-exclusion column and eluted directly into the SAXS flow-through capillary cell. Data were analyzed with Foxtrot (SOLEIL software group and SWING beamline) and the ATSAS software suite (EMBL, Hamburg, www.embl-hamburg.de/biosaxs/software.html). Scattered intensity from the atomic coordinates of the crystallographic structure was calculated using CRYSOL. The fit of the calculated intensity to the experimental intensity was assessed as described in [56].

## Supporting Information

Figure S1 **SDS-PAGE of purified recombinant proteins and characterization of complexes used in this study.** (A) SDS-PAGE analysis of purified recombinant Arf and BRAG proteins. (B) Formation of the Δ17Arf1–GDP/BRAG2^Sec7-PH/E498K^ intermediate analyzed by SEC-MALS. The molecular masses are 50.6±0.5 kDa for BRAG2^Sec7-PH/E498K^, 20.6±0.04 for Δ17Arf1–GDP, and 64.2±1.3 kDa for the complex. Size-exclusion chromatography coupled to multi-angle light scattering (SEC-MALS) analysis was performed essentially as described in [56] in a buffer containing 20 mM Hepes pH 7.4, 150 mM NaCl, and 20–30 µM of the proteins or complexes. (C) Formation of the nucleotide-free Δ17Arf1/BRAG2^Sec7-PH^ complex analyzed by size exclusion chromatography. The elution profiles of Δ17Arf1 (green), BRAG2^Sec7-PH^ (blue), and the nucleotide-free Δ17Arf1/BRAG2^Sec7-PH^ complex (red) are shown. The SDS-PAGE analysis of the Δ17Arf1/BRAG2^Sec7-PH^ peak is shown below. Note that BRAG2^Sec7-PH^ behaves as a monomer in size-exclusion chromatography. (D) BRAG2^Sec7-PH^ binds to liposomes containing PS and or PS and PI(4,5)P_2_. BRAG2^Sec7-PH^ was submitted to flotation assays using liposomes of the indicated composition (% of 1 mM total lipids). The 100% lane corresponds to the theoretical complete recovery of the protein in the fraction. k_obs_ measured with these liposome, and protein samples are as in Figure 3A. (E) Close-up view of the Arf/linker interface. Residues in contacts are given in Figure S3C.
(TIF)

Figure S2 **Sequence analysis of the linker and PH domain of BRAG2.** (A) Sequence alignment of the linker and PH domains of BRAG/IQSec/Schizo proteins from selected species. Invariant residues are in red. Human BRAG2 studied in this work is labelled IQEC1_human. Secondary structures observed in the BRAG2^Sec7-PH/E498K^ crystal structure are indicated. The invariant glutamate (E639) in strand β1 is indicated by a black arrowhead. Colored lines indicate the position of the Sec7 (magenta), the linker (yellow), and the PH domains (cyan). Residues located in the Sec7-PH linker/PH interface are indicated by a red arrowhead. Residues of the linker-PH tandem in contact with the Sec7 domain are indicated by a pink arrowhead. Residues of the linker in contact with Arf are indicated by cyan arrowheads. (B) Structure-based sequence alignment of BRAG2 with phospholipid-bound PH domains. Residues that can be structurally aligned with the structure of BRAG2 are in normal characters; residues that are nonsuperposable are in italics. Residues involved in binding lipid analogs were identified from the crystal structures using LIGPLOT (bold black characters). The highly conserved R654 in strand 2 mutated in this study is indicated in magenta. E639 of BRAG2 that replaces the invariant lysine in other PH domains is indicated in red. Positively charged residues of BRAG2 located at the periphery of the canonical lipid-binding pocket are indicated in cyan (see also Figure 3B). The crystal structures used in the alignment are: GRP1-IP4 (PDB code 2R0D), DAPP1-IP4 (PDB code 1FAO), Pleckstrin-IP5 (PDB code 2I5F), PEPP1-IP4 (PDB code 1UPR), AKT-PKB-IP4 (PDB code 1UNQ), PLC-IP3 (PDB code 1MAI), PDK1-IP4 (PDB code 1W1D), and Evectin-2-phosphoserine (PDB code 3AJ4).
(TIF)

Figure S3 **Intramolecular and intermolecular contacts of BRAG2.** (A) Intramolecular contacts between the linker and the PH domain. Contact maps were calculated with the Contact Map Analysis (CMA) server with a threshold of 10 Å^2^ [57]. (B) Intramolecular contacts between the linker-PH tandem and the Sec7 domain. (C) Intermolecular contacts between Arf1 and BRAG2^Sec7-PH^.
(TIF)

Figure S4 **Kinetics analysis of ^myr^Arf6 activation by BRAG2^Sec7-PH^.** (A) Representative tryptophan fluorescence kinetics of ^myr^Arf6 (0.4 µM) activation by BRAG2^Sec7-PH^ (0–1 nM range). Note the shape of the curves, which cannot be fitted by a single exponential. (B) Analysis of liposome polydispersity and radius by dynamic light scattering (DLS) along the exchange reaction. ^myr^Arf6 (0.4 µM), BRAG2 (1 nM), and GTP (100 µM) were added in sequence. DLS experiments were performed at 37°C in a DynaPro NanoStar apparatus (Wyatt technology) in HKM buffer in a disposable cuvette (Eppendorf). Data were analyzed using the software DYNAMICS (Wyatt Technology) assuming that the size distribution is a simple Gaussian function to yield the mean radius and polydispersity. Polydispersity and average radius were 29% and 100 Å for liposomes alone, 37% and 110 nm after addition of myrArf6–GDP, 33% and 114 nm after addition of BRAG2, and 27% and 111 nm after addition of GTP and completion of nucleotide exchange, ruling out that liposome aggregation occurs during the exchange reaction. (C) Analysis of initial velocities as a function of BRAG2^Sec7-PH^ concentration. The curves are linear and have similar slopes for ^myr^Arf1 and ^myr^ARF6.
(TIF)

Table S1 **Data collection and refinement statistics.**
(DOCX)

## Abbreviations

EGFR: epidermal growth factor receptor
GEF: guanine nucleotide exchange factor
PC: phosphatidylcholine
PE: phosphatidylethanolamine
PI: phosphatidylinositide
PS: phosphatidylserine
SAXS: small-angle X-ray scattering
