# Supplementary figures and images for: Integrated Conformational and Lipid-Sensing Regulation of Endosomal ArfGEF BRAG2
Source: PLoS Biol. 2013 Sep 10;11(9):e1001652. doi: 10.1371/journal.pbio.1001652 (PMC3769224; doi:10.1371/journal.pbio.1001652)

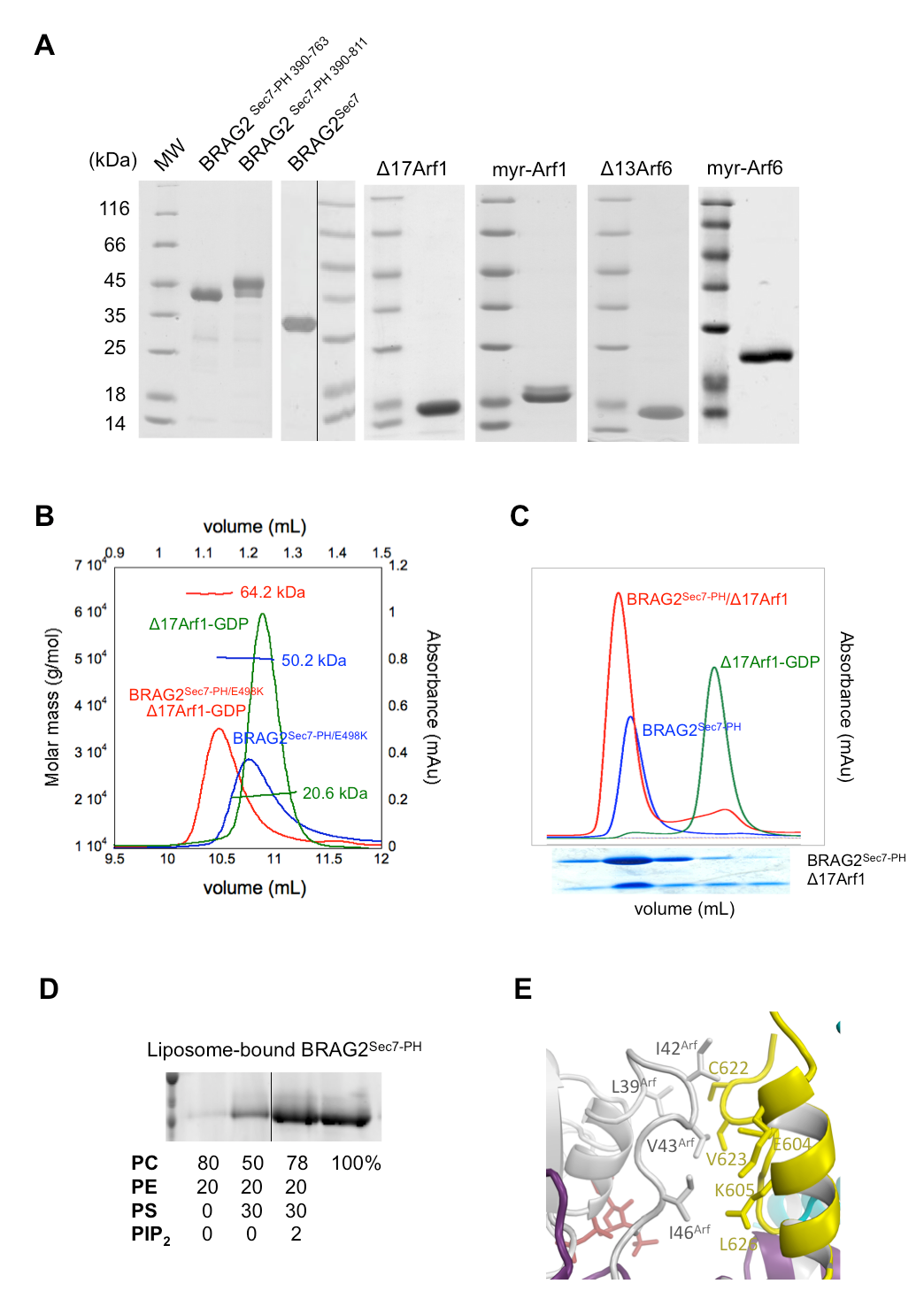

Supplement: Figure S1 — SDS-PAGE of purified recombinant proteins and characterization of complexes used in this study. (A) SDS-PAGE analysis of purified recombinant Arf and BRAG proteins. (B) Formation of the Δ17Arf1–GDP/BRAG2Sec7-PH/E498K intermediate analyzed by SEC-MALS. The molecular masses are 50.6±0.5 kDa for BRAG2Sec7-PH/E498K, 20.6±0.04 for Δ17Arf1–GDP, and 64.2±1.3 kDa for the complex. Size-exclusion chromatography coupled to multi-angle light scattering (SEC-MALS) analysis was performed essentially as described in [56] in a buffer containing 20 mM Hepes pH 7.4, 150 mM NaCl, and 20–30 µM of the proteins or complexes. (C) Formation of the nucleotide-free Δ17Arf1/BRAG2Sec7-PH complex analyzed by size exclusion chromatography. The elution profiles of Δ17Arf1 (green), BRAG2Sec7-PH (blue), and the nucleotide-free Δ17Arf1/BRAG2Sec7-PH complex (red) are shown. The SDS-PAGE analysis of the Δ17Arf1/BRAG2Sec7-PH peak is shown below. Note that BRAG2Sec7-PH behaves as a monomer in size-exclusion chromatography. (D) BRAG2Sec7-PH binds to liposomes containing PS and or PS and PI(4,5)P2. BRAG2Sec7-PH was submitted to flotation assays using liposomes of the indicated composition (% of 1 mM total lipids). The 100% lane corresponds to the theoretical complete recovery of the protein in the fraction. kobs measured with these liposome, and protein samples are as in Figure 3A. (E) Close-up view of the Arf/linker interface. Residues in contacts are given in Figure S3C. (TIF) [file pbio.1001652.s001.tif]

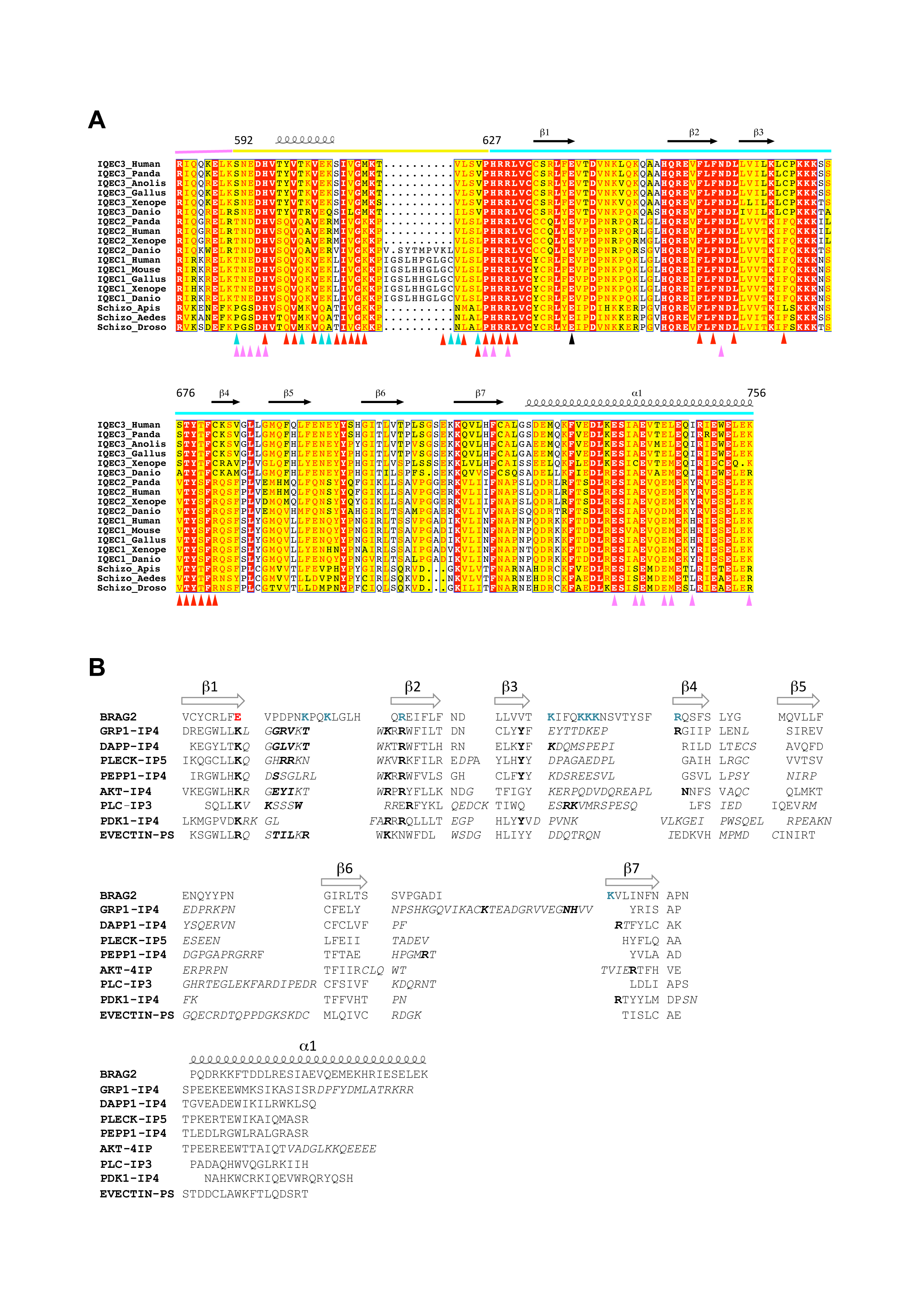

Supplement: Figure S2 — Sequence analysis of the linker and PH domain of BRAG2. (A) Sequence alignment of the linker and PH domains of BRAG/IQSec/Schizo proteins from selected species. Invariant residues are in red. Human BRAG2 studied in this work is labelled IQEC1_human. Secondary structures observed in the BRAG2Sec7-PH/E498K crystal structure are indicated. The invariant glutamate (E639) in strand β1 is indicated by a black arrowhead. Colored lines indicate the position of the Sec7 (magenta), the linker (yellow), and the PH domains (cyan). Residues located in the Sec7-PH linker/PH interface are indicated by a red arrowhead. Residues of the linker-PH tandem in contact with the Sec7 domain are indicated by a pink arrowhead. Residues of the linker in contact with Arf are indicated by cyan arrowheads. (B) Structure-based sequence alignment of BRAG2 with phospholipid-bound PH domains. Residues that can be structurally aligned with the structure of BRAG2 are in normal characters; residues that are nonsuperposable are in italics. Residues involved in binding lipid analogs were identified from the crystal structures using LIGPLOT (bold black characters). The highly conserved R654 in strand 2 mutated in this study is indicated in magenta. E639 of BRAG2 that replaces the invariant lysine in other PH domains is indicated in red. Positively charged residues of BRAG2 located at the periphery of the canonical lipid-binding pocket are indicated in cyan (see also Figure 3B). The crystal structures used in the alignment are: GRP1-IP4 (PDB code 2R0D), DAPP1-IP4 (PDB code 1FAO), Pleckstrin-IP5 (PDB code 2I5F), PEPP1-IP4 (PDB code 1UPR), AKT-PKB-IP4 (PDB code 1UNQ), PLC-IP3 (PDB code 1MAI), PDK1-IP4 (PDB code 1W1D), and Evectin-2-phosphoserine (PDB code 3AJ4). (TIF) [file pbio.1001652.s002.tif]

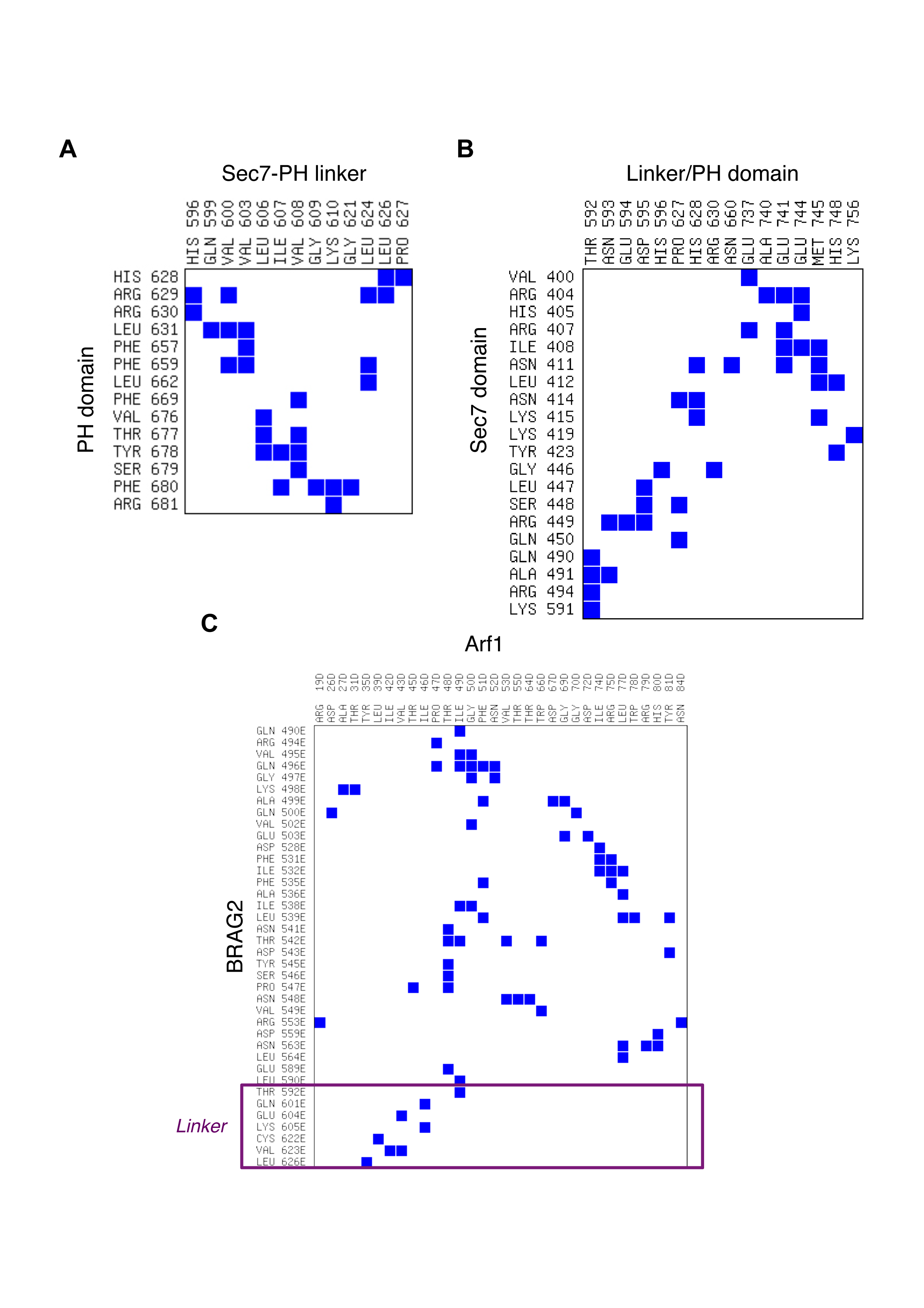

Supplement: Figure S3 — Intramolecular and intermolecular contacts of BRAG2. (A) Intramolecular contacts between the linker and the PH domain. Contact maps were calculated with the Contact Map Analysis (CMA) server with a threshold of 10 Å2 [57]. (B) Intramolecular contacts between the linker-PH tandem and the Sec7 domain. (C) Intermolecular contacts between Arf1 and BRAG2Sec7-PH. (TIF) [file pbio.1001652.s003.tif]

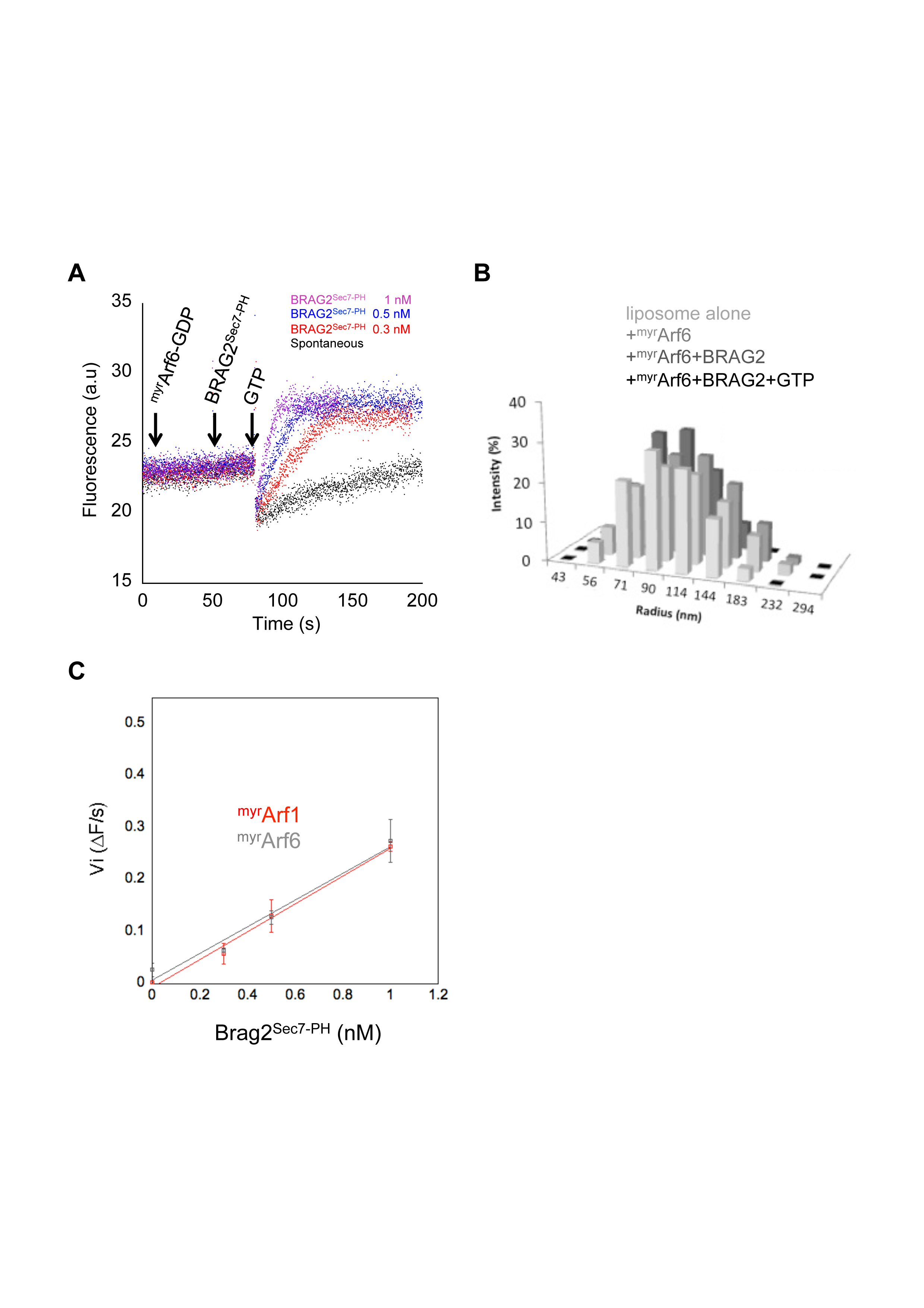

Supplement: Figure S4 — Kinetics analysis of myrArf6 activation by BRAG2Sec7-PH. (A) Representative tryptophan fluorescence kinetics of myrArf6 (0.4 µM) activation by BRAG2Sec7-PH (0–1 nM range). Note the shape of the curves, which cannot be fitted by a single exponential. (B) Analysis of liposome polydispersity and radius by dynamic light scattering (DLS) along the exchange reaction. myrArf6 (0.4 µM), BRAG2 (1 nM), and GTP (100 µM) were added in sequence. DLS experiments were performed at 37°C in a DynaPro NanoStar apparatus (Wyatt technology) in HKM buffer in a disposable cuvette (Eppendorf). Data were analyzed using the software DYNAMICS (Wyatt Technology) assuming that the size distribution is a simple Gaussian function to yield the mean radius and polydispersity. Polydispersity and average radius were 29% and 100 Å for liposomes alone, 37% and 110 nm after addition of myrArf6–GDP, 33% and 114 nm after addition of BRAG2, and 27% and 111 nm after addition of GTP and completion of nucleotide exchange, ruling out that liposome aggregation occurs during the exchange reaction. (C) Analysis of initial velocities as a function of BRAG2Sec7-PH concentration. The curves are linear and have similar slopes for myrArf1 and myrARF6. (TIF) [file pbio.1001652.s004.tif]
