# Supplementary material for: Integrated Conformational and Lipid-Sensing Regulation of Endosomal ArfGEF BRAG2
Source: PLoS Biol. 2013 Sep 10;11(9):e1001652. doi: 10.1371/journal.pbio.1001652 (PMC3769224; doi:10.1371/journal.pbio.1001652)
Supplement: Table S1 — Data collection and refinement statistics. (DOCX) [file pbio.1001652.s005.docx]

**Table S1.** **Data collection and refinement statistics**

|  | Δ17Arf1-GDP/  BRAG2^Sec7-PH/E298K^  native with His-tag | Δ17Arf1-GDP/  BRAG2^Sec7-PH/E298K^  Se-Met without His-tag |
| --- | --- | --- |
| **Data collection** |  |  |
| Wavelength | 0.98 | 0.98 |
| Space group | *C2* | *P2* |
| Cell dimensions |  |  |
| *a*, *b*, *c* (Å) | 210.72, 66.22, 91.06 | 90.98, 65.78, 196.86 |
| α, β, γ (°) | 90.00, 108.19, 90.00 | 90.00, 96.13, 90.00 |
| Resolution (Å) | 49.5 – 3.2 (3.8 – 3.2) * | 42.8 – 3.3 (3.4 – 3.3) |
| *R*_sym_ | 22.8 (42.4) | 20.1 (73.5) |
| *I* / σ*I* | 3.1 (1.9) | 6.8 (1.94) |
| Completeness (%) | 98.58 (97.7) | 99 (95.6) |
| Redundancy | 3.5 (2.5) | 3.24 (3.16) |
|  |  |  |
| **Refinement** |  |  |
| Resolution (Å) | 49.5 – 3.2 (3.37 – 3.2) | 42.8 – 3.3 (3.4 – 3.3) |
| No. reflections | 19,486 | 35,125 |
| *R*_work_ / *R*_free_ | 25.23 / 31.09 | 19.24 / 24.99 |
| No. atoms |  |  |
| Protein | 8,433 | 16,859 |
| *B*-factors (Å^2^) |  |  |
| Protein | 93.87 | 68.64 |
| R.m.s. deviations |  |  |
| Bond lengths (Å) | 0.009 | 0.01 |
| Bond angles (°) | 1.15 | 1.27 |
| Ramachandran plot |  |  |
| Residues in favoured regions (%) | 87.65 | 88.62 |
| Residues in allowed regions (%) | 9.53 | 8.58 |
| Outliers (%) | 2.82 | 2.8 |

*Values in parentheses are for highest-resolution shell. R_sym_=∑ (| I(h,i)-I(h) |))/∑ (I(h,i)
